# Image denoising method integrating ridgelet transform and improved wavelet threshold

**DOI:** 10.1371/journal.pone.0306706

**Published:** 2024-09-06

**Authors:** Bingbing Li, Yao Cong, Hongwei Mo

**Affiliations:** 1 College of Intelligent Systems Science and Engineering, Harbin Engineering University, Harbin, China; 2 Technology School, Jilin Business and Technology College, Changchun, China; Victoria University, AUSTRALIA

## Abstract

In the field of image processing, common noise types include Gaussian noise, salt and pepper noise, speckle noise, uniform noise and pulse noise. Different types of noise require different denoising algorithms and techniques to maintain image quality and fidelity. Traditional image denoising methods not only remove image noise, but also result in the detail loss in the image. It cannot guarantee the clean removal of noise information while preserving the true signal of the image. To address the aforementioned issues, an image denoising method combining an improved threshold function and wavelet transform is proposed in the experiment. Unlike traditional threshold functions, the improved threshold function is a continuous function that can avoid the pseudo Gibbs effect after image denoising and improve image quality. During the process, the output image of the finite ridge wave transform is first combined with the wavelet transform to improve the denoising performance. Then, an improved threshold function is introduced to enhance the quality of the reconstructed image. In addition, to evaluate the performance of different algorithms, different densities of Gaussian noise are added to Lena images of black, white, and color in the experiment. The results showed that when adding 0.010.01 variance Gaussian noise to black and white images, the peak signal-to-noise ratio of the research method increased by 2.58dB in a positive direction. The mean square error decreased by 0.10dB. When using the algorithm for denoising, the research method had a minimum denoising time of only 13ms, which saved 9ms and 3ms compared to the hard threshold algorithm (Hard TA) and soft threshold algorithm (Soft TA), respectively. The research method exhibited higher stability, with an average similarity error fluctuating within 0.89%. The above results indicate that the research method has smaller errors and better system stability in image denoising. It can be applied in the field of digital image denoising, which can effectively promote the positive development of image denoising technology to a certain extent.

## 1. Introduction

With the development of the times, digital images have become one of the ways for people to obtain important information from their daily lives. However, in image propagation and information acquisition, the noise can affect the image blurring, distortion, and even loss of important information [1, 2]. This also means that the quality of digital images will directly affect the accuracy of information transmission in the image, so it is necessary to perform certain denoising processing on the image before using digital images [3]. How to denoise images has become a difficult problem, which has attracted the attention of many scholars. Scholars have proposed statistical filtering and adaptive filtering methods to denoise images. In addition, Khmag A et al. proposed an image denoising method based on improved wavelet transform (WT) to improve the main structure of the original model image. This method could remove noise from natural images corrupted by Gaussian white noise and compress image signals. The results showed that the performance of this algorithm was significantly better than other algorithms [4]. Khmag A et al. proposed to implement pre-classification using clustering based on invariant moments and hidden Markov models to capture the wavelet transform dependence between additive Gaussian white noise pixels and their neighbors. Experimental results show that this method has great advantages in terms of peak signal-to-noise ratio and structural similarity under higher noise levels [5]. At the same time, some scholars have proposed that traditional methods such as standardization, enhancement (randomization), and data aggregation training can be used to overcome the standardization risks and modal differences in traditional denoising methods. However, when using these methods, it cannot be guaranteed that the obtained images can retain complete image information. The flourishing development of artificial intelligence technology provides a new direction for the innovation of image denoising technology. Hard threshold algorithm (Hard TA), and Soft threshold algorithm (Soft TA) are commonly used threshold processing functions in signal processing. The Hard TA refers to the threshold that cannot be exceeded by the detected data. The Soft TA refers to specifying a range of changes for the detected data. Semi-soft threshold algorithm (Semi-Soft TA) is a combination of Soft TA and Hard TA, which can maintain continuity near the threshold point, but there is a slope below the threshold point. The above three parameters play a crucial role in the effectiveness of image denoising, which can be collectively referred to as the improved threshold function. The improved threshold function is a continuous function that can effectively remove the interference of noise in images during image processing. This experiment proposes an image denoising method that integrates an improved threshold function and WT, aiming to removing noise information from the image and fully preserving the image information.

The contribution of this research is mainly in two aspects. Firstly, aiming at the problems existing in the traditional image denoising algorithm when dealing with complex noise types, it adopts the fused improved threshold function and WT, and combined with WT and ridge algorithm. Therefore, the denoising algorithm can better adapt to images with different noise types. Secondly, the setting method of threshold algorithm is improved, which further improves the denoising quality in the image denoising.

The research has four parts. The first is a literature review on image denoising methods that integrate improved threshold functions with WT. It mainly introduces the application of the improved threshold and WT algorithm in other fields, as well as the research history of image denoising. The second part has two sections. The first section mainly introduces the core algorithm of the fusion algorithm and the method of obtaining key parameters. The second section introduces the optimization methods of the fusion algorithm and the running ideas of the algorithm. The third part has two sections. The first section discusses the denoising performance of the fusion algorithm in different demand environments such as color images, black and white images, and fingerprint images. The second section mainly introduces the stability, computational speed, and application performance of the algorithm in complex environments with fused noise. The fourth part summarizes the first three parts, and analyzes the specific performance of the fusion algorithm and the shortcomings in the research process.

## 2. Related work

WT, as a change analysis method, can effectively remove the correlation between different extracted features. It has fast computing speed, which is favored by scholars. S. Ramakanth and other scholars designed a new algorithm based on continuous WT to achieve automatic target recognition using jet engine modulated radar signals. It is applied to simulated signals affected by noise. Coiflet and complex Morlet wavelets are used for research. Compared with the improved Hilbert Huang Transform, the improved algorithm has better performance [6]. Khmag A et al. also proposed a nonlinear filtering method based on a two-step switching scheme to eliminate salt-and-pepper noise and additive Gaussian white noise [7]. Khmag A and Ramli’s team also used a combination of wavelet denoising algorithm and statistical principal component analysis algorithm to denoise images. This algorithm integrates (PCA) to exploit the subjective and objective quality of the observed images produced by the filtering process [8]. Y. Xu et al. used an improved empirical WT initialized multi-scale fluctuation dispersion entropy method to study the characteristics in active distribution network (ADN) models under grid connection, interruption, and islanding conditions. Disturbance signals in high permeability ADN were detected and classified. The research results indicated that the improved method had robustness and good accuracy [9]. Gao L et al. proposed a signal denoising method that combined mathematical morphology and wavelet adaptive threshold to accurately diagnose ECG signals. The signal-to-noise ratio was used to adjust the threshold parameters, and mathematical morphology was used to eliminate low-frequency noise. Then an innovative denoising method was obtained. The results showed that the root mean square difference and signal-to-noise ratio of this algorithm were significantly improved. The denoising effect was significant [10].

As the mainstream way of information dissemination nowadays, digital image transmission has received a lot of attention and research on the noise processing. Khare S K et al. used adaptive tunable Q-WT for automatic selection when studying emotional recognition of EEG signals. Grey Wolf Optimization was applied to obtain the optimal tuning parameters. Compared with traditional methods, the improved method had strong advantages in accuracy, computational speed, and other aspects [11]. Qiao W et al. adopted a combination prediction model based on WT, short-term memory, and stacked automatic encoder when dealing with strategic planning issues in power generation. The research results indicated that the model had higher prediction accuracy and faster prediction speed [12]. FU Q et al. proposed an image denoising method based on an improved threshold function to eliminate the deviation between different threshold functions. The improved threshold function was combined to process low-frequency noise and reconstruct the image. The results showed that the PSNR of this algorithm was significantly improved by about 5%. The mean square error (MSE) was significantly reduced, and the performance was superior [13]. M. Begum’s team proposed a digital image encryption method based on discrete cosine transform and wavelet transform to ensure the security of images during network transmission. During the process, Arnold mapping is used to encrypt the transmitted image, followed by generating the image through multiple operations. The results indicate that the improved method has good performance and can effectively enhance security [14]. H. Xu et al. adopted a new method based on structural matrix restoration on reducing noise in hyperspectral images. The research results indicated that it exceeded other methods in visual and quantitative indicators such as PSNR and spectral angular distance (SAD) [15].

In summary, high noise levels have a negative impact on image quality, resulting in blurred image details, unclear edges, etc., thereby reducing the image perception quality. It may also cause the effective information in the image to be submerged or destroyed, and even lead to important details in the image can not be accurately identified and utilized. Image denoising has high research value. Currently, a large number of scholars have participated in the research on this issue. However, few scholars have combined improved threshold functions with wavelet algorithms to solve image denoising. Therefore, an image denoising method combining improved threshold function and WT is used to improve the quality of reconstructed images. Combining the improved threshold function with WT algorithm, it is expected to improve the denoising ability of the algorithm by combining the excellent performance of the two algorithms in image denoising. The improved threshold function can better adapt to the noise variety of the image, and can adjust the relevant parameters to adjust the threshold selection range according to the actual situation. The WT algorithm greatly improves the denoising ability of the fusion algorithm by segmenting the image.

## 3. Design of image denoising methods

In the image denoising, the threshold parameters will affect the denoising performance. Combining ridge wave algorithm with wavelet algorithm, and absorbing the characteristics of Hard TA and Soft TA, an TA is proposed to obtain the parameters required in the image denoising.

### 3.1. The main structure design of denoising algorithm

**Fig 1:**
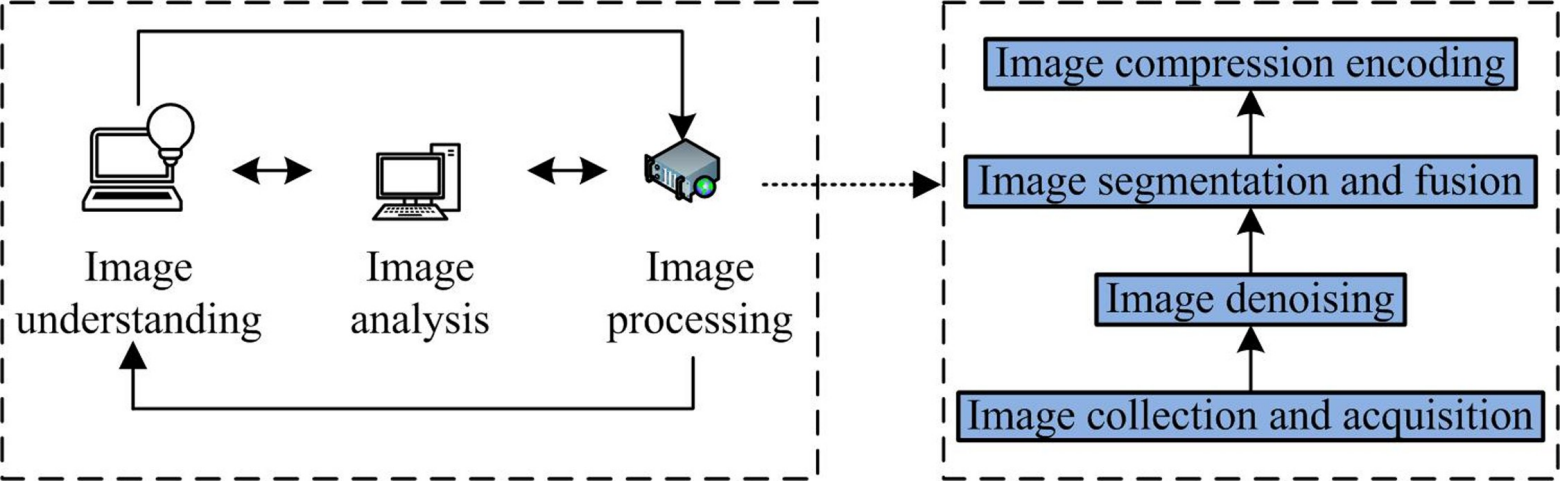
Correlation between image denoising and image processing.

During the process of obtaining and transmitting images, they are easily affected by the environment, leading to the doped noise in the output image. The signal of the image itself is mixed with noise signals, resulting in blurred image details. This can cause problems such as image information loss during the decoding [16]. Therefore, the removal of image noise has received widespread attention from scholars for a long time. The noise in images is reduced by integrating improved threshold algorithms with wavelet algorithms. A brief introduction is given to the optimization ideas and parameter selection of improved threshold algorithm and wavelet algorithm. The noise processing of images is the foundation of image processing. The relationship between the two is shown in Fig 1.

**Fig 2:**
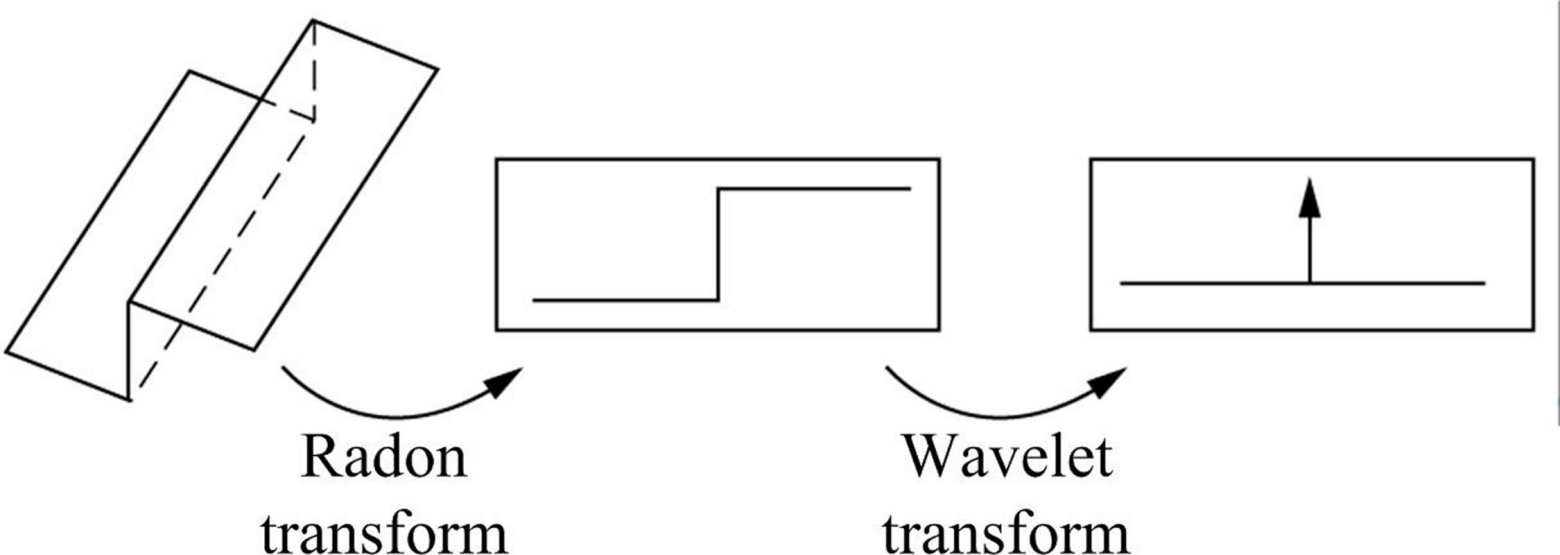
Ridge wave transformation flowchart.

In Fig 1, the relationship between image denoising and image processing can be divided into three parts, namely image understanding, image analysis and image processing. The three parts complement each other and are related to each other. Image processing can be mainly divided into four steps. Firstly, the images are collect and obtain. Then the selected technology is used to denoise the image. Different image information is segmented and relevant information is fused. Finally, image processing is implemented. Compression and encoding are used to achieve image denoising effect. The denoised image can better reflect the objective data and real information of the original image, providing strong support for image analysis and understanding processes. The WT is extensively applied in the image denoising, which has good denoising effects. However, traditional wavelet algorithms still have discontinuity at the threshold point of the Hard TA. The Soft TA has drawbacks such as deviation from the real signal. Therefore, improvements are made to the threshold and threshold function [17]. The output image of the finite ridge transform and the improved threshold wavelet algorithm are combined to improve the denoising performance. The specific flowchart of ridge wave transformation is shown in Fig 2.

In Fig 2, the ridgelet transform can be mainly divided into two steps, namely Radon transform and WT. Specifically, after obtaining an original image, the subspace domain image is used to undergo ridgelet transformation to obtain the image ridgelet coefficients (containing noise). Then, the coefficients higher than the threshold are retained. The coefficients lower than the threshold are removed. The ridgelet transform of the final image is inversely transformed into a spatial domain image. If the transformed image has warping distortion, it needs to be removed by Vienna transformation. The experiment assumes that *ψ*(*t*) represents a one-dimensional wavelet, then the two-dimensional ridgelet can be expressed as Eq (1).


$$\psi_{a,b,\theta }=a^{-1/2}\psi ((x_{1}\cos\theta +x_{2}\sin\theta -b)/a)$$ (1)

In Eq (1), $a,b\in R,a\neq 0,x=(x_{1},x_{2})$. *θ* is the directional parameter. This equation is relatively complex. Therefore, the expression of two-dimensional ridge waves is transformed to reduce the mathematical difficulty. The modified two-dimensional function ridge wave transformation is shown in Eq (2).


$$CRT_{f}(a,b,\theta )=\int_{R^{2}}\psi_{a,b,\theta }(x)f(x)dx$$ (2)

In Eq (2), the conic curve function is converted into an integral. *f*(*x*) is the weight parameter change function in the direction of *y*. The continuous Radon transformation of the two-dimensional function can be obtained by the two-dimensional ridge-wave transformation. The continuous Radon transform and discrete form of the two-dimensional ridge-wave transform can be expressed, as shown in Eq (3).


$$\left\{\begin{array}{l} R_{f}(\theta ,t)=\int_{R^{2}}f(x)\delta (x_{1}\cos\theta +x_{2}\sin\theta -t)dx\\ R\left(n,m\right)=|F\left(n,m\right)|-|F\left(n-1,m\right)|-|F\left(n+1,m\right)|-|F\left(n,m-1\right)|-|F\left(n,m+1\right)| \end{array}\right.$$ (3)

In Eq (3), *R*_*f*_(*θ*,*t*) represents the continuous Radon transformation of the two-dimensional ridge-wave transformation. *t* is the time parameter. After obtaining the Radon transformation expression of the two-dimensional function ridge transform expression, the form can be rewritten to obtain the expression of the two-dimensional ridge transform. *R*(*n*,*m*) represents the discrete value of the two-dimensional ridge-wave transform result. *F*(*n*,*m*) represents the transformation result of the two-dimensional discrete function in the frequency domain. The discrete form of the ridge transform of the two-dimensional function indicates that the discrete value R (n,m) of the ridge transform result is equal to the amplitude value of the corresponding position in the frequency domain minus the amplitude value of the adjacent positions above and below [18]. This formula can be used to detect the structure of the ridged wave in the two-dimensional function. By calculating the discrete value of the ridged wave transform result, the ridged sign and edge information in the function can be found. The specific form of a two-dimensional ridge wave is shown in Eq (4).


$$\psi_{a,b,\theta }=\int_{R}\psi_{a,b}(t)\delta (x_{1}\cos\theta +x_{2}\sin\theta -t)dt$$ (4)

In Eq (4), $\psi_{a,b}(t)=a^{-1/2}\psi ((t-b)/a)$. *ψ*_*a*,*b*_(*t*) is the Fourier transform form of one-dimensional ridge waves. The one-dimensional ridged wave in the form of Fourier transform refers to the spectrum obtained after the one-dimensional signal is Fourier transformed, which represents the distribution of the signal in the frequency domain [19]. One-dimensional ridged wave transform based on Fourier transform frequency domain analysis method, by calculating the spectrum amplitude and phase information of the signal, the frequency characteristics and periodic structure of the signal can be revealed. One-dimensional ridge transform can highlight the ridge structure and suppress other frequency components by Fourier transform and spectrum processing, such as threshold processing or spectrum amplitude filtering, and can extract the ridge structure and edge information in the signal. The result obtained by substituting two-dimensional ridged wave into the two-dimensional functional ridged wave transform is shown in Eq (5).


$$CRT_{f}(a,b,\theta )=\int_{R^{2}}\int_{R}\psi_{a,b}(t)f(x)\delta (x_{1}\cos\theta +x_{2}\sin\theta -t)dtdx$$ (5)

In Eq (5), the final ridge transformation expression can be obtained by performing Radon transformations on the obtained transformation expressions. The specific form of this expression is shown in Eq (6).


$$CRT_{f}(a,b,\theta )=\langle \psi_{a,b}(t),R_{f}(\theta ,t)\rangle =\int_{R}\psi_{a,b}(t)R_{f}(\theta ,t)dt$$ (6)

In Eq (6), *ψ*(⋅) is a projection of the one-dimensional wavelet in the *θ* direction. The ridge wave transformation process is as follows. The transformation method obtained by performing Radon transform first and then WT processing. The image size *I*(*i*,*j*) is *p* × *p*. *p* represents a prime number. The transformation definition of finite Radon is shown in Eq (7).


$$r_{k}[l]=\frac{1}{\sqrt{p}}\sum_{(i,j)\in L_{k,l}}I(i,j)$$ (7)

In Eq (7), *L*_*k*,*l*_ represents a straight line. The slope is k and the intercept is l. This line is shown in Eq (8).


$$\left\{\begin{array}{l} L_{k,l}=\left\{(i,j):j=ki+l(\mathrm{mod}p),i\in Z_{p}\right\},k\in Z\\ L_{p.l}=\left\{(i,j):j\in Z_{p}\right\} \end{array}\right.$$ (8)

In Eq (8), the finite Radon transform is considered as the sum of pixel values of lines in different directions. *r*_*k*_(*l*) is the matrix of ridge wave transformation. The image has multiple blocks. It is expressed as a term in the matrix. There are p+1 directions in the matrix. Each direction covers p pixel values. Therefore, the ridge transform matrix can cover the entire pixel of the image, and then perform WT on each column in the matrix, resulting in finite ridge coefficients. By performing Radon transform and WT on the initial image, the processed image is obtained. Among them, the inverse transform signal obtained through finite Radon transform is a very important part of the signal reconstruction process. This part needs to be obtained through finite ridge inverse transformation. The definition of the finite back projection operator is shown in Eq (9).


$$\left\{\begin{array}{l} I(i,j)=\frac{1}{\sqrt{p}}\sum_{(k,l)\in P_{i,j}}r_{k}(l),(i,j)\in Z_{p}^{2}\\ P_{i,j}=\left\{(k,l):l=j-ki(\mathrm{mod}p)k\in Z_{p}\right\}\cup \left\{\left(p,i\right)\right\} \end{array}\right.$$ (9)

In Eq (9), *I*(*i*,*j*) stands for the pixel value at the corresponding coordinate of the image. *r*_*k*_(*l*) stands for the finite Radon coefficient. Based on this method, the finite back projection operator is restored to the original image. The finite Radon transform uses modular p-operation, which sometimes leads to the "wrapping" in the image. The output image shows streaks, which affect the output image quality. Therefore, the main improvement direction for the ridge algorithm is to reduce the surround phenomenon of the algorithm and improve the quality of the output image.

### 3.2. Optimization of fusion algorithm

**Fig 3:**
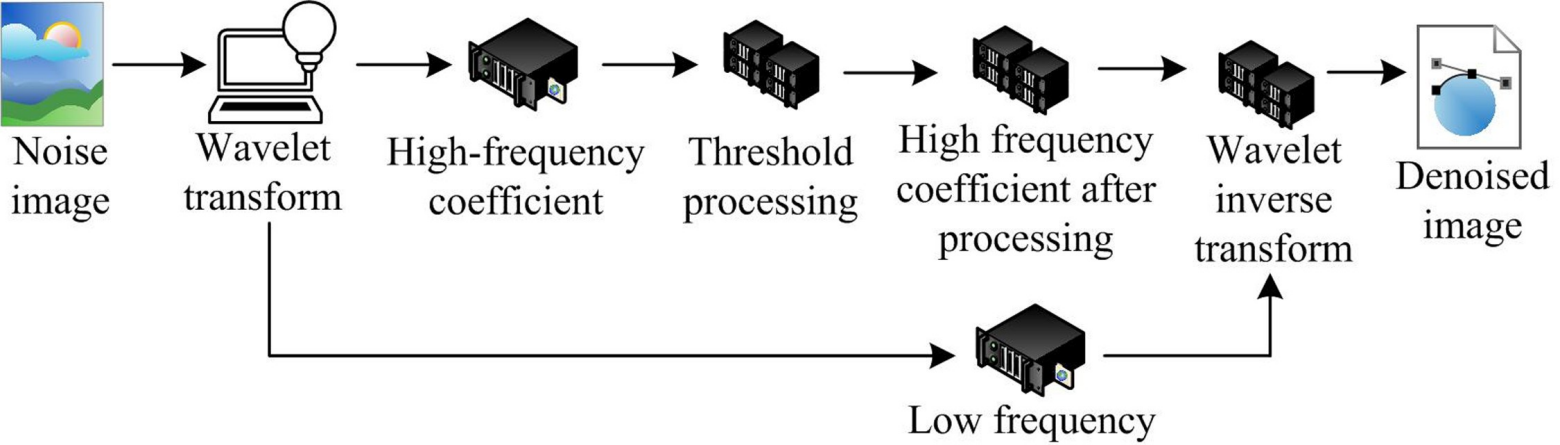
Flow of wavelet threshold denoising.

Threshold denoising method of WT is widely used in signal and image processing. WT has the characteristics of multi-scale analysis, which can provide information in both frequency domain and time domain. This gives it an advantage in capturing local details and overall trends. In addition, the WT can compress the signal, remove redundant information and retain the main features. The WT mainly achieves denoising by setting a threshold. The WT coefficients that are less than the threshold are considered noise. The remaining coefficients are retained as effective signals. It has the simple principle, small computational complexity, and good denoising effect. Fig 3 displays the specific denoising process.

In Fig 3, WT decomposes the signal to obtain wavelet coefficients. The obtained wavelet coefficients are subjected to threshold processing to obtain an estimated value [20]. The difference between the two is minimized by continuously adjusting the threshold. Then, the estimated value is reconstructed through inverse WT to obtain the estimated signal, which is the original denoised signal. The signal is f(t)=s(t)+n(t). *s*(*t*) refers to the original signal. *n*(*t*) refers to the noise. Taking this signal as an example, discrete sampling is performed on the signal to obtain the discrete signal. The WT coefficients of *f*(*t*) can be obtained. The specific form is shown in Eq (10).


$$W_{f}(j,k)=2^{\frac{j}{2}}\sum_{n=0}^{N-1}f(n)\psi (2^{j}n-k)$$ (10)

In Eq (10), the calculation method for *W*_*f*_(*j*,*k*) is relatively complex. *ψ*(*t*) is the analytical expression. Therefore, the WT coefficients are transformed through a dual scale equation. The original coefficient of change is transformed, as shown in Eq (11).


$$\left\{\begin{array}{l} S_{f}(j+1,k)=S_{f}(j,k)*h(j,k)\\ W_{f}(j+1,k)=S_{f}(j,k)*g(j,k) \end{array}\right.$$ (11)

In Eq (11), *h* stands for the low-pass filter of the scaling function *φ*(*t*). *g* is a high-pass filter for the *ψ*(*t*). *S*_*f*_(0,*k*) stands for the initial signal. *S*_*f*_(*j*,*k*) stands for the approximation coefficient on the *j* scale. *W*_*f*_(*j*,*k*) stands for the wavelet coefficient. After obtaining a recursive implementation method for the coefficient, the equation is reconstructed. The specific form of the reconstructed WT is shown in Eq (12).


$$S_{f}(j-1,k)=S_{f}(j,k)*\tilde{h}(j,k)+W_{f}(j,k)*\tilde{g}(j,k)$$ (12)

In Eq (12), the coefficient is ω_*j*,*k*_. After performing discrete WT of signal *f*(*k*), the coefficients consist of two parts. *s*(*k*) stands for the real signal, corresponding to the coefficient *W*_*s*_(*j*,*k*). The noise *n*(*k*) corresponds to the coefficient *W*_*n*_(*j*,*k*). At this point, the construction of WT equation is completed. After obtaining the denoised image output by WT, ridge wave transform is used to denoise the image. The outputs of the last two algorithms are fused to improve the denoising effect. The denoising method using ridge transform is similar to WT. Namely, threshold processing is performed through finite ridge coefficients to get ridge transformation coefficients obtained from noise and signal transformation. Ridged denoising is applied to reduce image noise and improve signal-to-noise ratio. The specific process is as follows. Firstly, the image is preprocessed, and the image size is adjusted to the prime size. Next, the image is decomposed by finite ridge transform and the ridge coefficient is obtained. Then, the ridge coefficient is filtered by setting the threshold to remove the noise in the image. Finally, the processed ridged coefficients are synthesized into reconstructed images by using the inverse transformation of finite ridged waves. After the reconstructed image is obtained, the image can be processed using the Wiener filter to reduce the surround effect that may be caused by the ridge denoising, which further enhances the image quality. In the wavelet threshold denoising, setting the threshold value is a very important step, which will directly affect the quality of the image after denoising. The classical threshold selection methods include universal threshold method, SUREShrink threshold method, heuristic threshold method and minimax threshold method. The most extensively applied method is the universal threshold. The selected method is simple, which has been widely used in practical life. After determining the threshold selection method, the TA is used to obtain the threshold. Among them, Hard TA and Soft TA are the most common methods. The specific expression is shown in Eq (13).


$$\left\{\begin{array}{l} {\hat{\omega }}_{j,k}=\left\{\begin{array}{l} \omega_{j,k},|\omega_{j,k}|\geq T\\ 0,|\omega_{j,k}|<T \end{array}\right.\\ {\hat{\omega }}_{j,k}=\left\{\begin{array}{l} \mathrm{sgn}(\omega_{j,k})(\omega_{j,k}-T),|\omega_{j,k}|\geq T\\ 0,|\omega_{j,k}|<T \end{array}\right. \end{array}\right.$$ (13)

**Fig 4:**
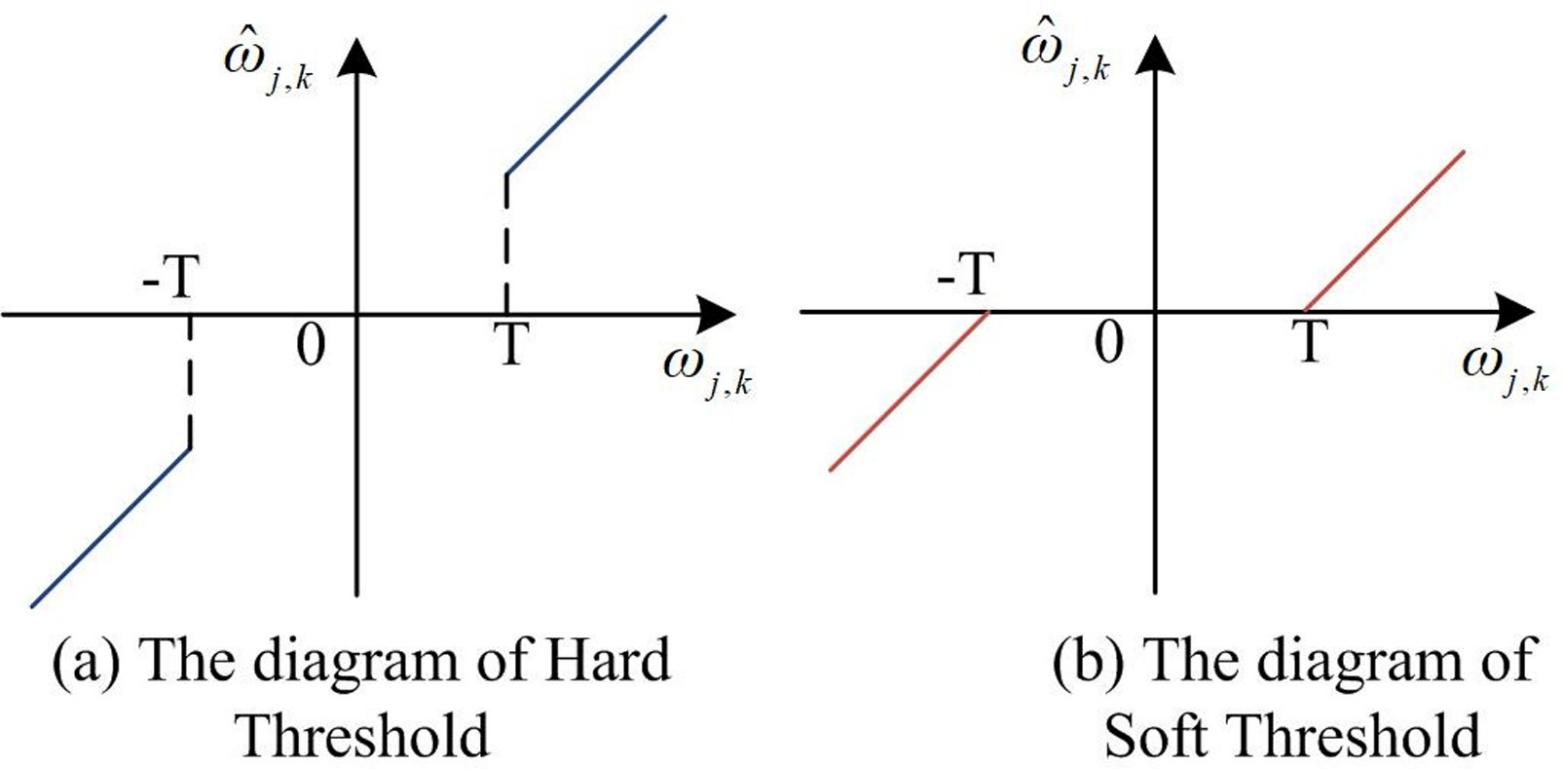
The diagram of Hard TA and Soft TA.

In Eq (13), ω_*j*,*k*_ stands for the wavelet coefficient. ${\hat{\omega }}_{j,k}$stands for the new coefficient after denoising. *T* refers to the threshold. The constructed Hard TA and Soft TA are displayed in Fig 4.

In Fig 4, each of the two TAs has the own advantages. For example, the denoising process of the Hard TA can better preserve the original features, while the denoising process of the Soft TA can make the signal smoother. Among them, Soft TA zeros signal components below a certain threshold, and scale the components that are greater than the threshold. Hard TA zeros signal components below a certain threshold and keeps components above that threshold unchanged. Semi-Soft TA is a combination of Soft TA and Hard TA. In Semi-Soft TA, the retained component larger than the threshold is scaled, while the component smaller than the threshold is zeroed directly. When facing the problem of image denoising with larger image scales, the energy amplitude of the noise decreases due to the larger image scale. The traditional universal threshold method to reduce noise can lead to excessive denoising, resulting in the information being removed as noise. The quality of denoised images decreases. Among them, the image reconstructed by the Hard TA generates pseudo Gibbs lines due to discontinuity at the threshold points, resulting in image oscillation. The Soft TA has a constant difference between the denoised signal and the real signal, which leads to blurring and quality degradation of the reconstructed output image. In order to overcome such shortcomings, a more effective and applicable TA is constructed based on the understanding of the limitations and shortcomings of traditional TA, in-depth understanding of mathematical principles, and in-depth thinking and exploration of practical problems, as shown in Eq (14).


$${\hat{\omega }}_{j,k}=\left\{\begin{array}{l} \omega_{j,k}+T-\frac{2T}{\exp({\left|\frac{T}{\omega_{j,k}}\right|}^{n}-1)+1+n},\omega_{j,k}\leq -T\\ \frac{2\mathrm{sgn}(\omega_{j,k}){\left|\omega_{j,k}\right|}^{n+1}}{\left[\exp({\left|\frac{\omega_{j,k}}{T}\right|}^{n}-1)+1+n\right]T^{n}},|\omega_{j,k}|<T\\ \omega_{j,k}-T+\frac{2T}{\exp({\left|\frac{T}{\omega_{j,k}}\right|}^{n}-1)+1+n},\omega_{j,k}\geq T \end{array}\right.$$ (14)

**Fig 5:**
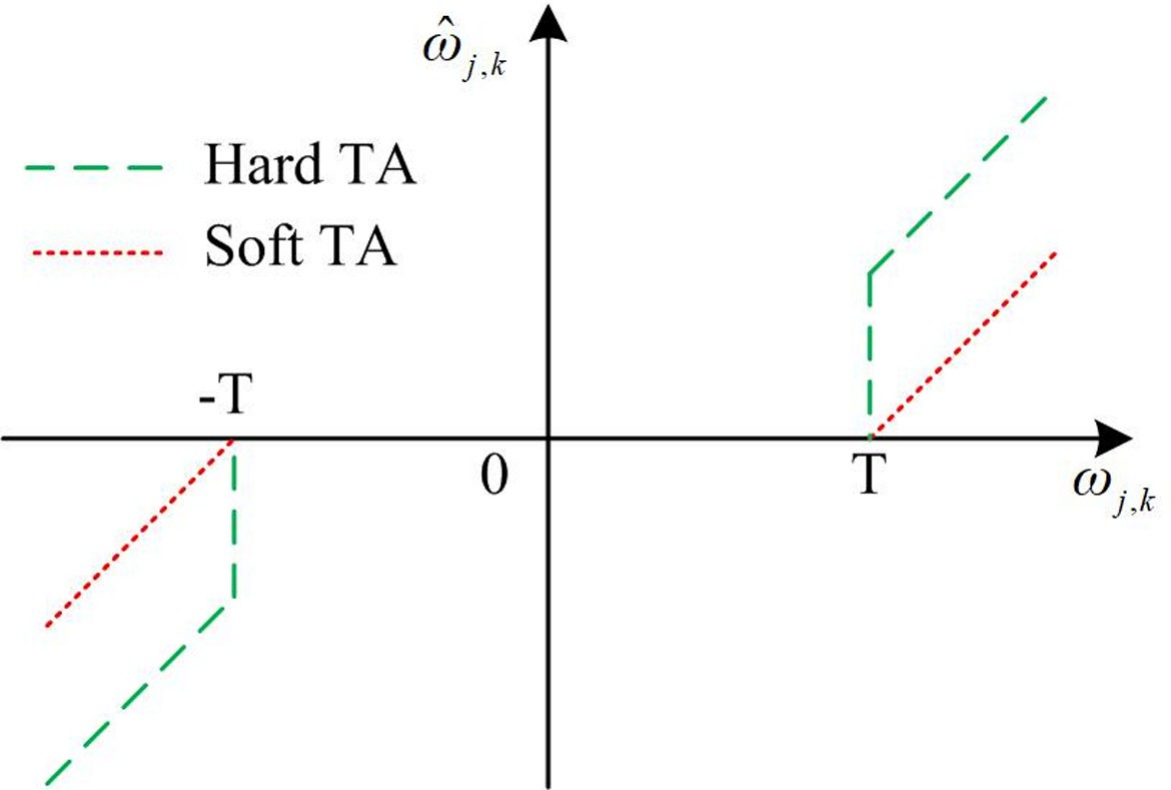
The improved Hard TA and Soft TA.

In Eq (14), *n* stands for the adjustment parameter. The improved TA combines the characteristics of Hard TA and Soft TA to enhance the flexibility of threshold changes. It has better noise reduction performance for larger scale images. The improved Hard TA and Soft TA are shown in Fig 5.

In Fig 5, the improved TA still maintains continuity at ±*T*. When adjusting parameter *n* = 0, the denoised wavelet coefficients will not change. When the adjustment coefficient approaches positive infinity, the form of the denoised wavelet coefficient is shown in Eq (15).


$${\hat{\omega }}_{j,k}=\left\{\begin{array}{l} \omega_{j,k}+T,\omega_{j,k}\leq -T\\ 0,|\omega_{j,k}|<T\\ \omega_{j,k}-T,\omega_{j,k}\geq T \end{array}\right.$$ (15)

**Fig 6:**
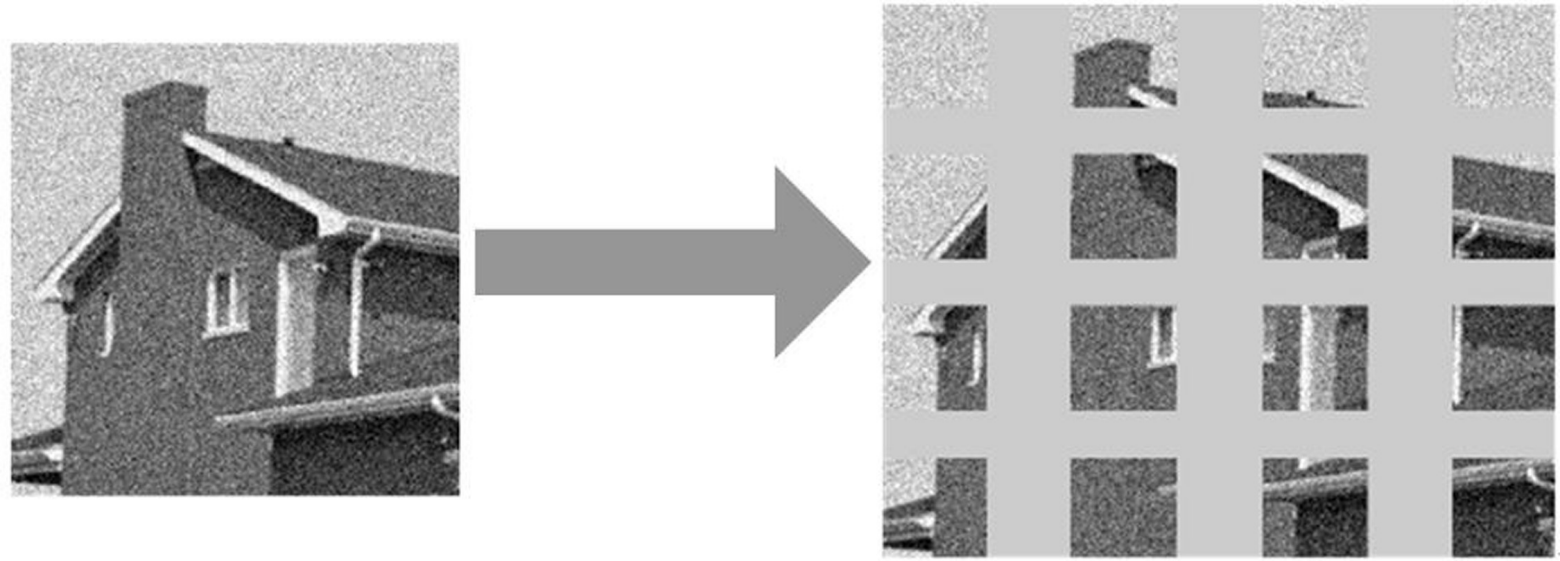
Block ridge wave denoising flowchart.

In Eq (15), the improved TA can change the wavelet parameters of the TA as the adjustment parameters change. Increasing the value of the adjustment parameter can gradually bring the TA closer to the Soft TA. Adjusting parameters not only makes the improved TA continuous, but also reduces the mathematical processing difficulty. It also adjusts the adjustability of the parameters. By repeatedly fitting, the optimal denoising effect is obtained through optimization. WT is highly effective in describing the singularity. However, there is a lack of ability to describe lines. Therefore, the WT in image denoising inevitably leads to blurring and distortion of the image. Ridge wave transform has better performance than WT in processing images, edges, or linear contours. Compared with WT, ridge transform can provide sparse representation of edges, thus better preserving the linear features of the image. The specific content of the segmented ridge denoising algorithm is shown in Fig 6.

**Fig 7:**
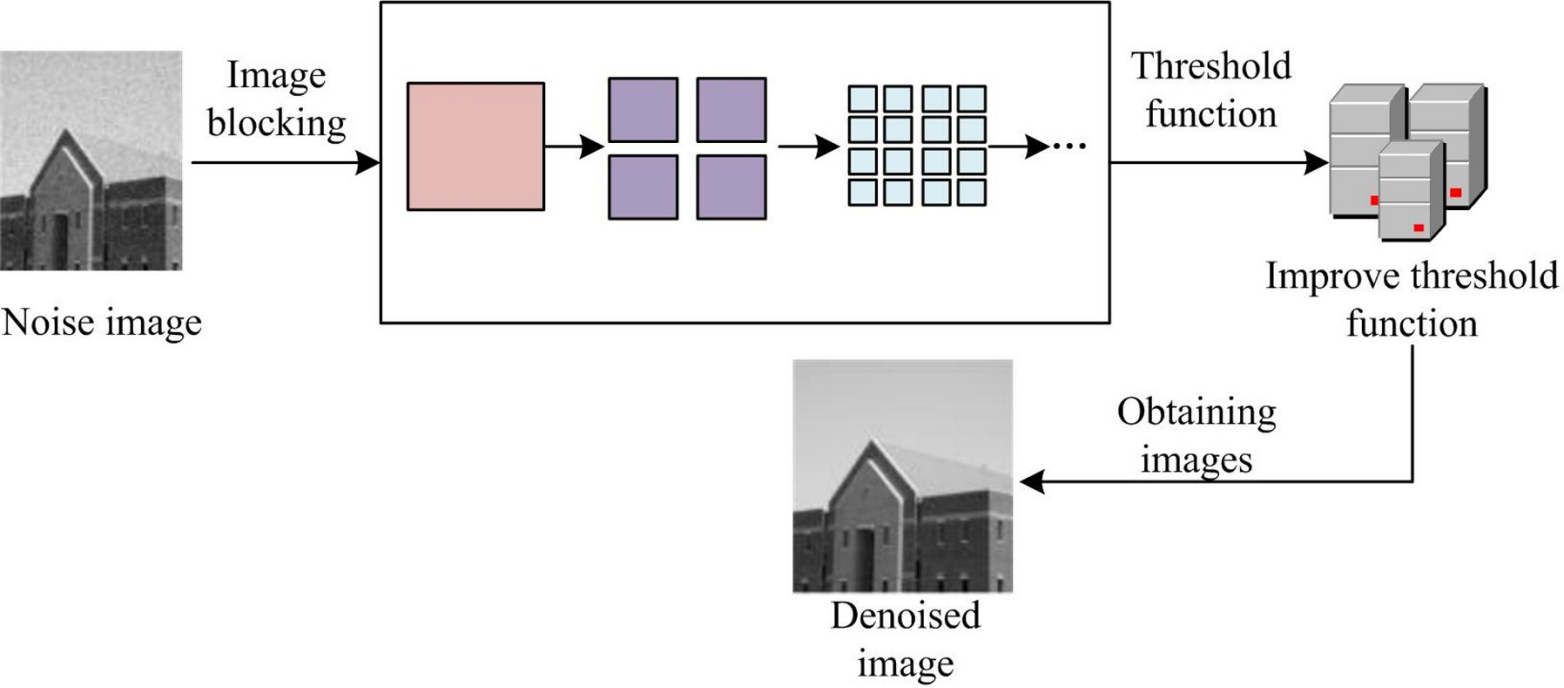
A hybrid algorithm based on improved threshold function and wavelet transform.

In Fig 6, the first step is to divide the image into equally sized square blocks. Then, the size of each small square block is converted to a prime size. The ridge wave transform decomposition is applied. A threshold is selected for denoising based on each square block. Finally, each square block is reconstructed using inverse ridge wave transform. The denoised square blocks are restored to their original size, and then concatenated to obtain a complete image. From this, the steps of the algorithm can be obtained, which includes a total of 7 steps. (1) The noise image of size N × N is divide into square image blocks of size b × B (b<N), so that these small image blocks do not overlap with each other. The position of each small square image block in the original image is recorded. (2) The bi-linear interpolation is reused to transform the square image block into p × P size (where p is the minimum prime greater than b). (3) The ridge wave transformation is performed on each square image block to retain the ridge wave coefficient matrix for each block. (4) A threshold is selected based on the ridge coefficient matrix of each square image block and the denoising processing is performed. (5) The processed ridge wave coefficients are subjected to inverse ridge wave transformation to preserve the processed square blocks and restore all square blocks to their original sizes. (6) These blocks are restored to their original positions and glued together to form a complete image. (7) Wiener filters are used to filter denoised images to reduce the influence of "surrounding" effects. To achieve better denoising results in image denoising tasks, the wavelet thresholding and ridge transform processing are combined. The specific fusion process is shown in Fig 7.

In Fig 7, the combination steps of wavelet threshold algorithm and ridge wave algorithm are as follows. Firstly, the noise is added to the original image. Then, the improved wavelet threshold method is applied to denoise the noisy image. It is applied to the segmented ridge wave transformation method for denoising noisy images. Finally, the denoised images obtained by the two methods are selected with appropriate wavelet bases for wavelet decomposition. The low-frequency and high-frequency wavelet coefficients are extracted, respectively. The high-frequency and low-frequency parts of two sets of coefficients are added and summed, and then the average value is taken to obtain a new set of high-frequency and low-frequency coefficients. Finally, by applying the inverse WT, the fused image can be obtained. When denoising images, the structure, smoothness and denoising time of the image will be affected to a certain extent. Therefore, the denoising time, structural changes of the image after denoising, image smoothness, and similarity error are studied. The performance of the four algorithms is compared with objective performance indicators (the calculation is very simple, and the experiment does not provide special calculation formulas here).

## 4. Result analysis

As one of the mainstream information dissemination methods, the research on denoising technology of digital image propagation has always received a lot of attention from scholars. To specifically reflect the performance of the image processing technology that combines the improved TA and WT, the improved algorithm is compared with traditional image denoising algorithms. The denoising performance in different images and noise situations is statistically analyzed to verify the superiority.

### 4.1. Performance analysis of algorithm

To verify the superior performance of the hybrid algorithm constructed in the experiment, the black and white and color Lena image data sets are selected as the experimental data set. The images contained in the Lena image dataset are rich in detail, smooth areas, shadows and textures, making them ideal for evaluating the performance of various image processing techniques [21]. The experiment selects a 512x512 pixel version of the Lena image, which can be either color (24-bit) or grayscale (8-bit). Images come from image databases of various academic institutions, such as the SIPi image database of the University of Southern California (USC), as well as public domain resources on the Internet.

To ensure the smooth progress of the experiment, all experiments are conducted in a unified simulation environment. The parameters are set as follows. The CPU is Intel Xeon Gold 6230 20-Core Processor, 2.1 GHz. The network is 10Gbps Ethernet. The storage is 1TB NVMe SSD. The server is Ubuntu Server 20.04 LTS. The development environment is Python. The image processing software is the MATLAB platform. The data management tool is PostgreSQL software. The system learning framework is PyTorch.

**Fig 8:**
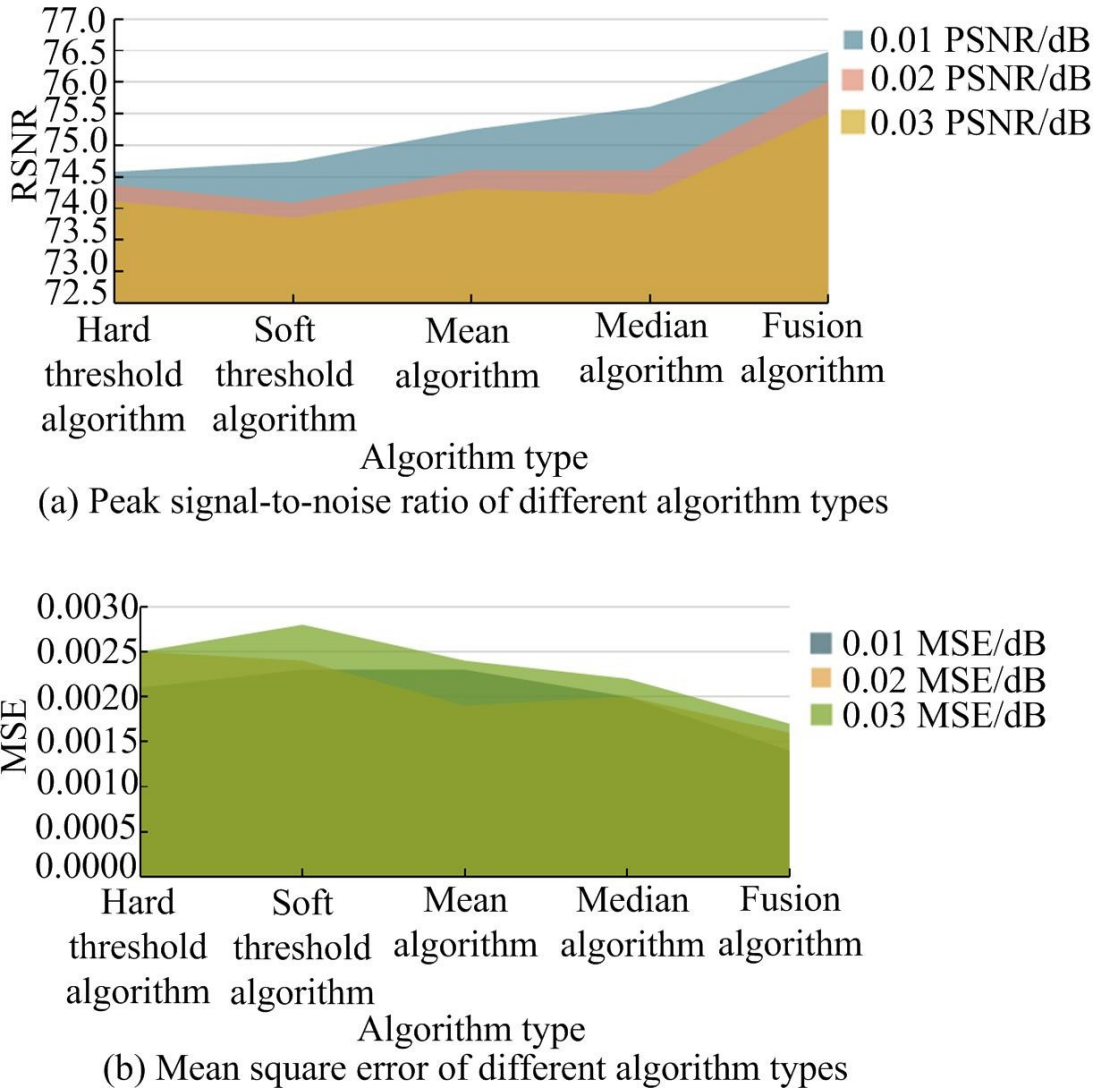
Comparison results of PSNR and MSE of gray images under different algorithms.

Gaussian noise with a variance of 0.01 is added to two test images in the experiment. Gaussian noise with large noise variance usually has poor denoising effect. A higher noise variance means that the noise signal strength is larger, which makes the noise more difficult to identify and separate in the original signal.Traditional algorithms such as HTA, STA, Semi-Soft TA, Mean algorithm (MA), Median algorithm (MeA), and improved threshold fusion wavelet algorithms are used for image denoising. The objective evaluation indicators are applied to assess the denoising effect of the image. Fig 8 displays the specific evaluation results.

**Fig 9:**
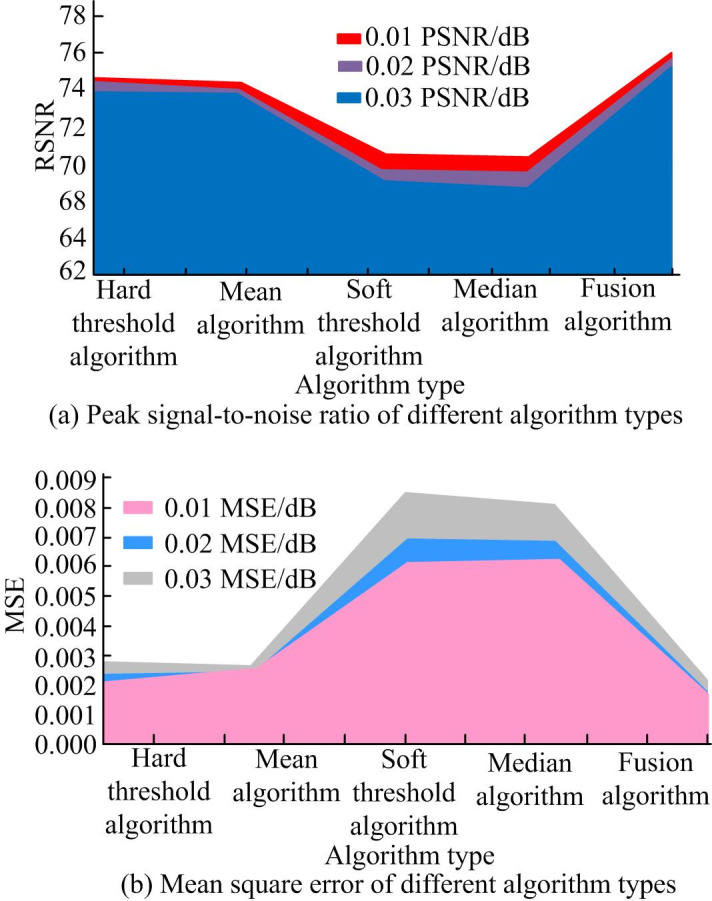
Comparison results of PSNR and MSE of color images under different algorithms.

In Fig 8, a black and white image with 0.01 variance Gaussian noise is denoised. The PSNR of the fusion algorithm increased by 2.58dB, 2.31dB, 1.98dB, 1.59dB, and 1.15dB compared to the HTA, STA, Semi-Soft TA, MA, and MeA, respectively. The MSE decreases by 0.05%, 0.10%, 0.04%, 0.06%, and 0.05%, respectively. When the amplitude of the noise increased to 0.03, the PSNR increased by 1.84%, 2.21%, 1.85%, 1.56%, and 1.71%. The MSE decreased by 0.07%, 0.10%, 0.05%, 0.05%, and 0.04%, respectively. The fusion algorithm has shown significant improvement in both PSNR and MSE values. The traditional methods and fusion algorithms are tested using color Lena images. The specific indicator gap is shown in Fig 9.

In Fig 9, when using different algorithms to process Gaussian noise with a difference of 0.01 in the image, the PSNRs of the fusion algorithm, Hard TA, Soft TA, Semi Soft TA, MA, and MeA were 74.25%, 71.70%, 71.94%, 72.26%, 72.66%, and 73.08%, respectively. The MSE of the fusion algorithm was 0.21. In addition, when the amplitude of Gaussian noise increased to 0.03, compared to traditional methods, the PSNR of the hybrid algorithm increased by 1.87%, 2.21%, 1.85%, 1.54%, and 1.68%, respectively. The MSE decreased by 0.06dB, 0.11dB, 0.05dB, 0.07dB, and 0.04dB, respectively. By comparing the above results, the PSNR of the fusion algorithm significantly increased, while the value of MSE significantly decreased. This also indicates that there is a small difference between the reconstructed image and the original image. The difference between the predicted and actual values is also small, resulting in better image quality. At the same time, the hybrid algorithm can be widely used for denoising operations on black and white images and color images. The denoising effect is very good.

### 4.2. Analysis of application effect of algorithm

**Fig 10:**
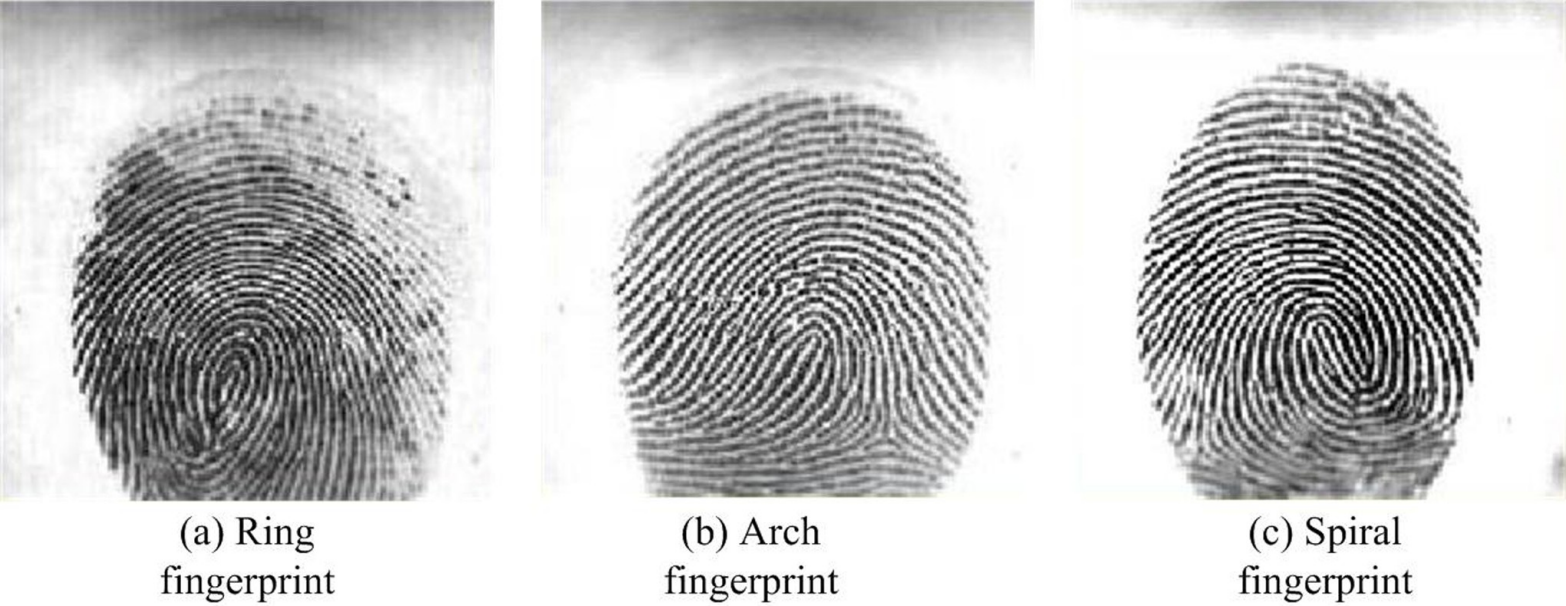
Three images of fingerprint patterns.

Fingerprint images are different from ordinary images. To comprehensively consider the performance of Fusion algorithm, three fingerprint patterns from the FCV2004 fingerprint database are selected as test images to measure the noise reduction performance of traditional algorithms and fusion algorithms. The images of three fingerprint patterns are shown in Fig 10.

All three fingerprint images are added with a mixture of Gaussian and salt and pepper noise, with a noise density of 0.01. The denoising evaluation indicators of traditional methods and fusion algorithms are displayed in Table 1.

**Table 1:** Denoising evaluation indicators for fingerprint images using different algorithms.

Image sequence number	Evaluating indicator/dB	Hard TA	Soft TA	Semi-Soft TA	Mean algorithm	Median algorithm	Fusion algorithm
1	PSNR	71.13	72.04	71.57	71.82	72.21	73.53
	MSE	0.0049	0.0037	0.0041	0.0039	0.0040	0.0024
	FOM	49.36	58.69	77.51	68.32	71.21	83.52
	MAE	4.53	3.34	2.96	3.68	2.36	1.25
2	PSNR	70.31	70.83	70.59	71.52	72.20	73.58
	MSE	0.0060	0.0051	0.0055	0.0041	0.0042	0.0031
	FOM	48.48	61.71	75.25	70.20	69.13	78.64
	MAE	4.32	3.18	3.01	3.02	2.58	1.18
3	PSNR	70.71	71.25	71.07	72.22	72.30	73.74
	MSE	0.0054	0.0050	0.0049	0.0038	0.0041	0.0023
	FOM	50.24	65.89	72.84	75.85	74.56	79.63
	MAE	3.96	2.93	3.28	3.27	2.81	1.27

Table 1 shows the denoising effect of fingerprint images with added noise. The fusion algorithm is compared with other algorithms. Taking the denoising effect of fingerprint images with serial number 1 as an example, the PSNR values increased by 3.35%, 2.01%, 2.68%, 2.51%, and 1.80%, respectively. The MSE decreased by 0.23%, 0.13%, 0.21%, 0.13%, and 0.11%, respectively. For sequence 1, the FOM of the Hard TA and Soft TA was 49.36 and 58.69, respectively, while the FOM of the fusion algorithm was 83.52, indicating that the fusion algorithm had better overall performance compared to the Hard TA and Soft TA. For sequence 1, the MAE of the Soft TA was 3.34, while the fusion algorithm was 1.25, which showed that the fusion algorithm could obtain higher prediction accuracy when denoising the fingerprint image. The fusion algorithm still shows excellent performance for mixed noise mixed in fingerprint images. This is mainly because the experimental design method introduces Hard TA and Soft TA. The results are obtained after calculation. The values of the evaluation indicators obtained after denoising are better than those of several other algorithms, proving that the image denoising method that combines the improved threshold algorithm and the WT algorithm can maintain high denoising performance when processing single noise or mixed noise images. After studying the impact of image color and noise type on the performance of denoising algorithms, from the structural similarity of images, the denoising performance of different algorithms is studied. The specific evaluation indicators are shown in Table 2.

**Table 2:** Structural changes of images after denoising using different algorithms.

Evaluating indicator	MSE		PSNR		SSIM		References
Image sequence number	1	2	1	2	1	2	
Hard TA	120.731	191.251	27.313	25.315	0.973	0.917	[22]
Soft TA	146.748	187.004	26.46	25.412	0.967	0.913	[23]
Semi-Soft TA	119.317	172.758	27.364	25.756	0.974	0.922	[3]
Mean algorithm	96.077	146.607	28.305	27.138	0.979	0.935	[24]
Fusion algorithm	80.457	125.687	29.075	27.138	0.982	0.945	This study

**Fig 11:**
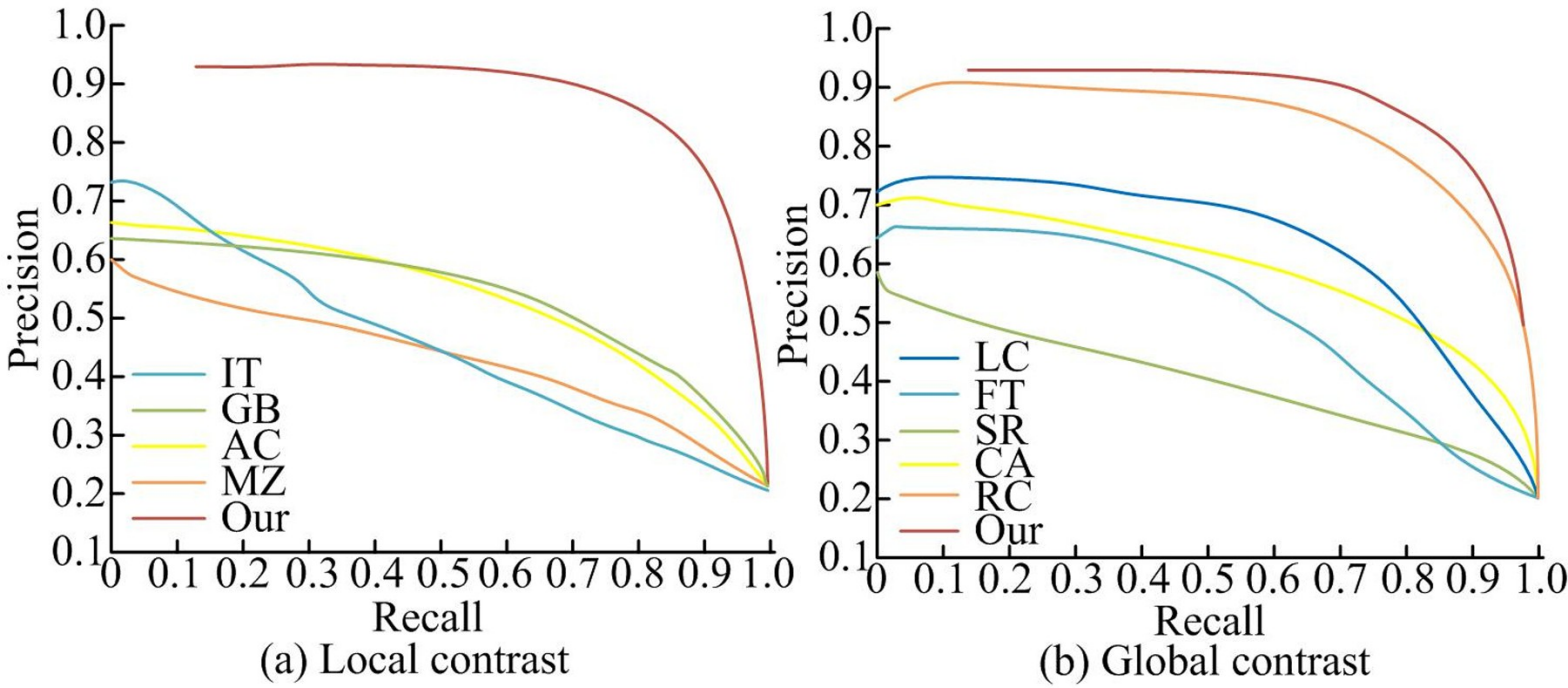
Image smoothness of different methods.

In Table 2, taking the image with serial number 1 as an example, the fusion algorithm outperformed the Hard TA, Soft TA, Semi Soft TA, and MA by 0.973, 0.967, 0.922, and 0.979, respectively. By comparison, the fusion algorithm had the smallest MSE, the highest PSNR, and the highest structural similarity. This also indicates that the details of the denoised image obtained by using fusion algorithms are more fully preserved. The image contours are smoother, and the image clarity is also higher. To explore the smoothness of the fusion algorithm’s output images, nine classic detection algorithms are selected. 1000 natural images are selected from the standard test image library MSRA1000 to group and calculate the smoothness of the output images. The study statistically analyzes the denoising performance and image smoothness of various methods by manually marking the results. The precision and recall of each algorithm are also calculated. The P-R curve was plotted. The specific P-R curves are shown in Fig 11.

**Fig 12:**
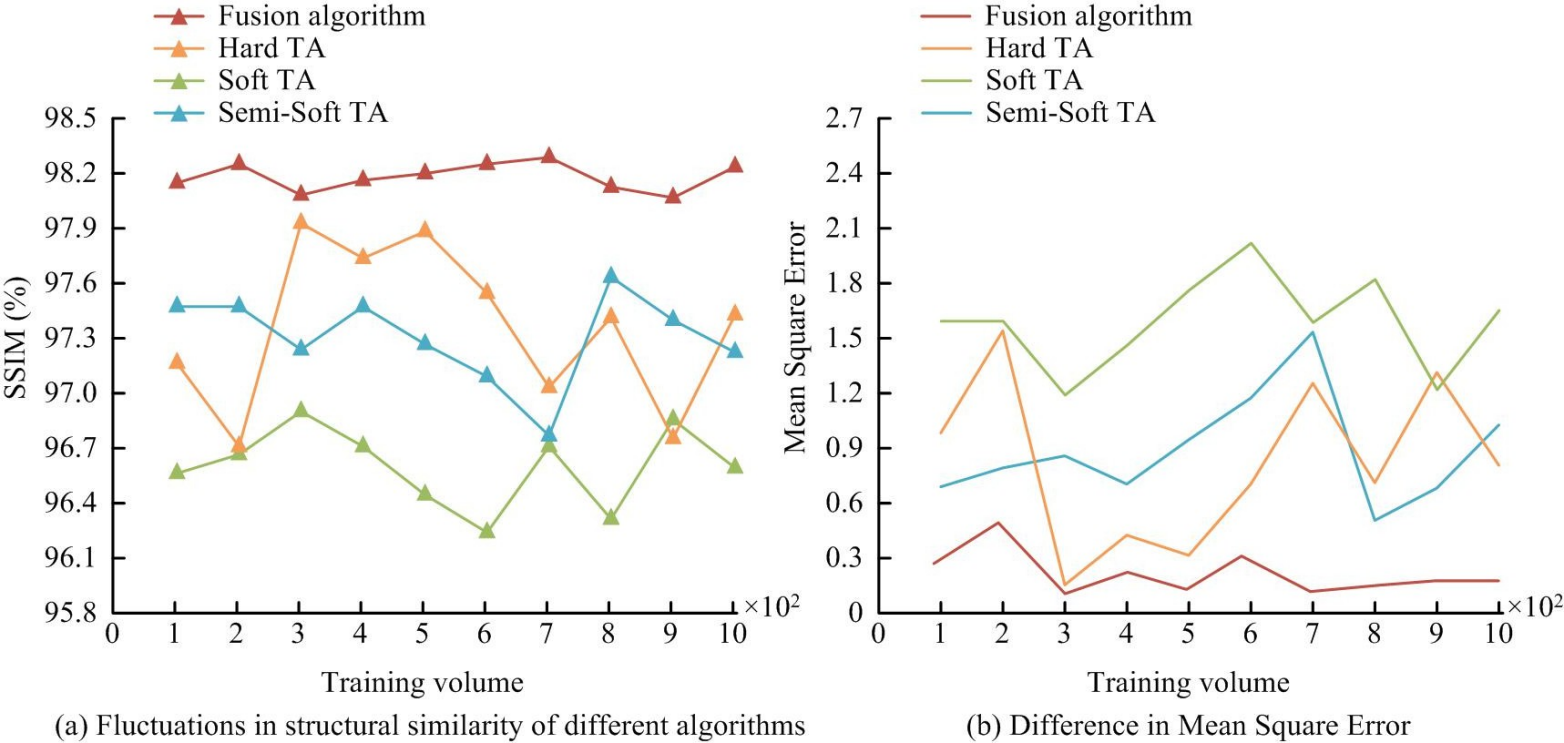
The SSIM for the different algorithms.

In Fig 11, in addition to the RC algorithm based on global contrast, the fusion algorithm, whether in local or global contrast, exceeded the traditional methods. The P-R curve also shows that the Fusion algorithm has smoother changes, stable image output performance, and significant performance improvement than existing methods. Meanwhile, in different application environments, the noise density of the target image that denoising algorithms face varies. Traditional HTA, STA, Semi-Soft TA, and fusion algorithms are selected for comparison. The MSRA1000 data set is selected as the task data set. This data set is a standard test gallery for visual saliency detection, covering a variety of scenes, such as natural scenery, urban street scenes, indoor environments, etc. This data set is provided by Microsoft Research Asia, MSRA, containing 1,000 high-quality color images and their corresponding saliency annotations. These images are divided into 10 levels, with 100 images as one level, gradually increasing the number of tests. The average structural similarity of each level is calculated. The specific situation obtained is shown in Fig 12.

**Fig 13:**
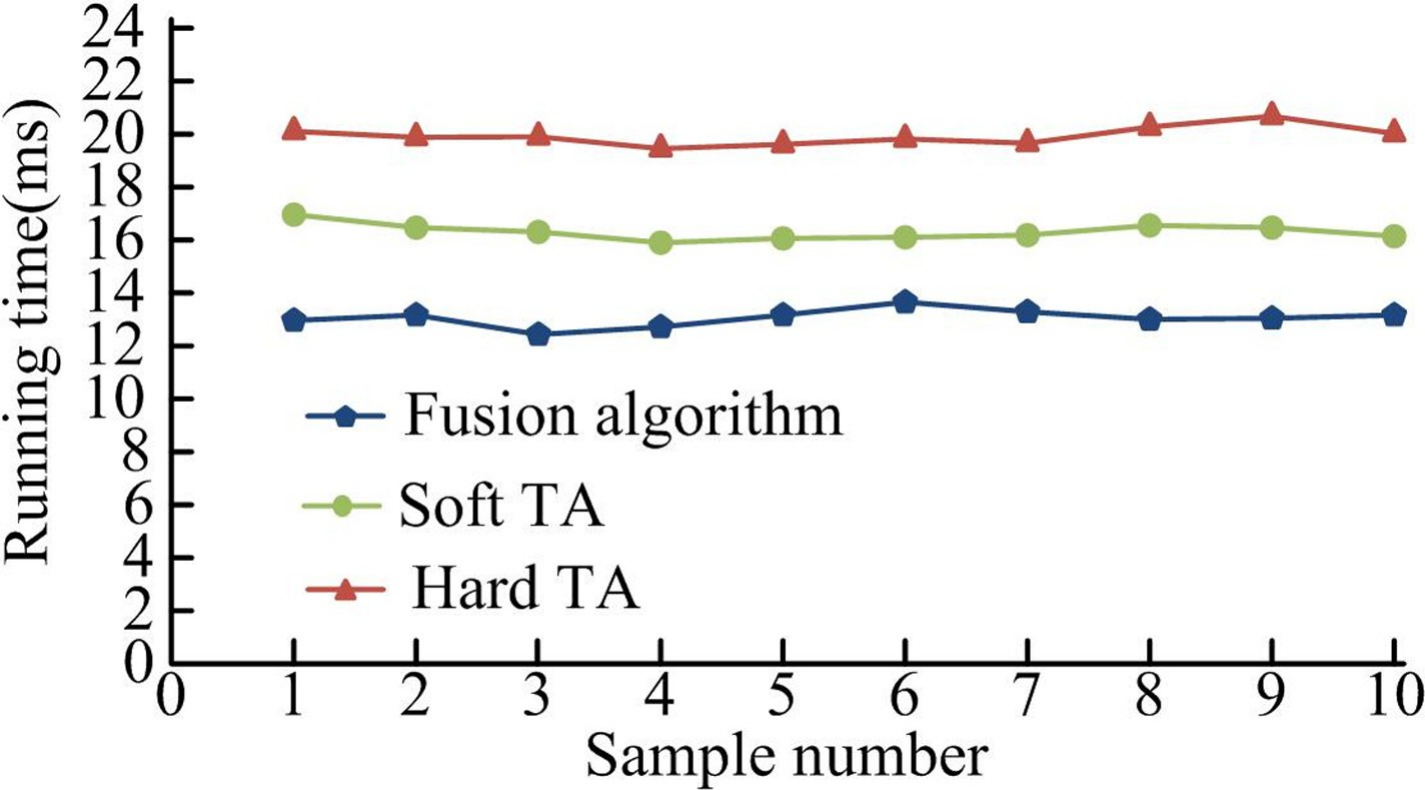
Time spent on image denoising using different algorithms.

Fig 12(A) shows the SSIM values obtained by different algorithms. As the amount of data changes, the SSIM values of all algorithms change with different amplitudes. During the entire process of changing the amount of data, the SSIM value of the fusion algorithm remained near 98.3%. The SSIM values of other algorithms were significantly less than 98.3%. Fig 12(B) shows the MSE values obtained by different algorithms running on the MSRA database data set. When the amount of data reached 300 images, the fusion algorithm had the smallest MSE, with a value of 0.114. At this time, the MSE of Hard TA and Soft TA were greater than the MSE of the fusion algorithm, with values of 0.158 and 1.361, respectively, much larger than the fusion algorithm. From the above results, the image obtained by using the fusion algorithm had a high SSIM value and a low MSE value, that is, the distortion of the obtained image is small, and the denoised image is closer to the clear image. The difference is smaller. After examining the robustness of the algorithm, it is also necessary to analyze the calculation time of the algorithm. The MSRA1000 images in the standard test gallery are used as the test set. The 100 natural images are divided into 10 groups. The groups are denoised and statistically denoised. The time spent in the process and the time spent on denoising by different methods are shown in Fig 13.

**Fig 14:**
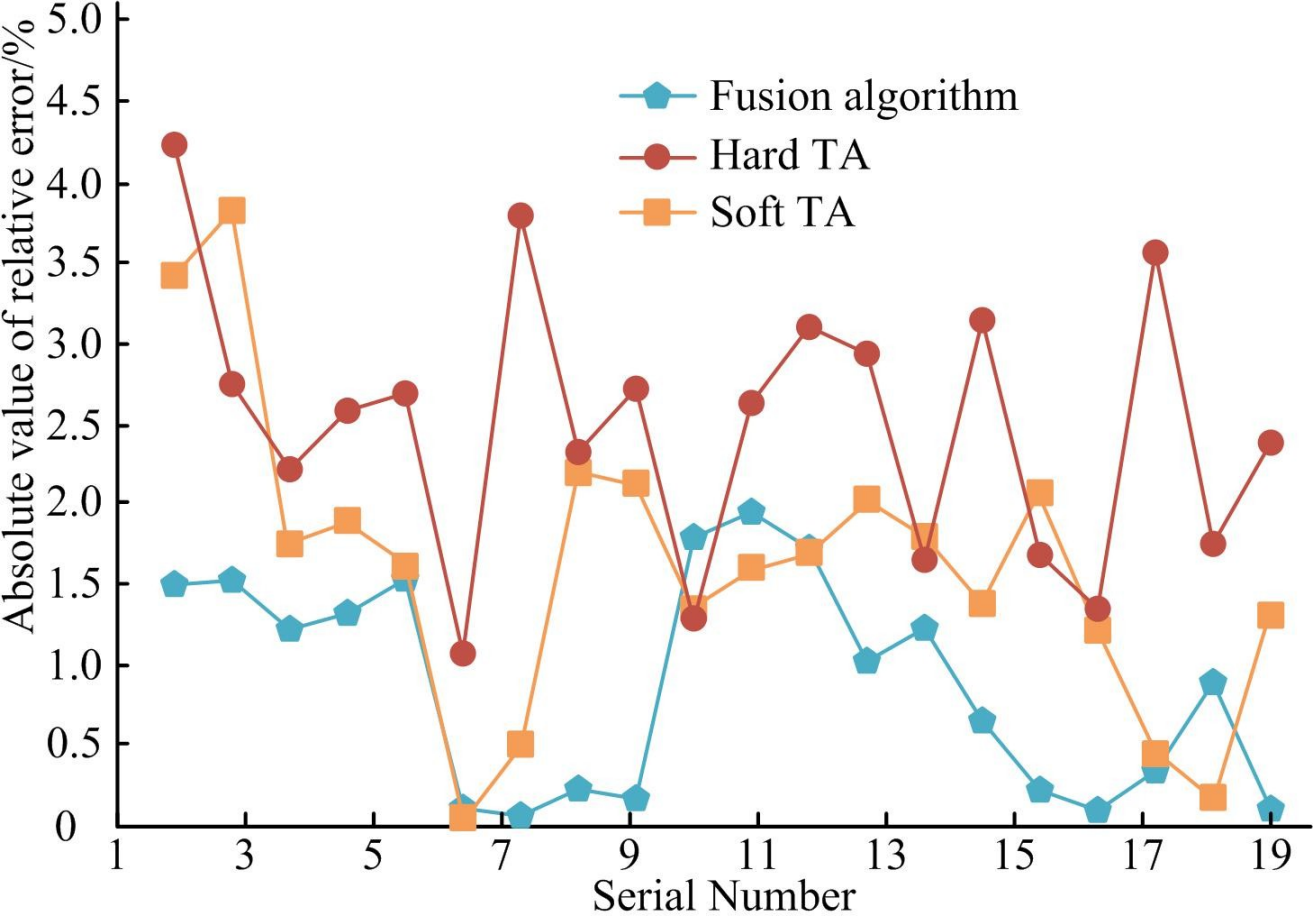
Similarity error of different methods.

In Fig 13, the fusion algorithm performs well in denoising time consumption. Among them, the minimum denoising time was only 13ms. Compared to the HTA and the STA, it saved 9ms and 3ms respectively. The improved algorithm combining threshold algorithm and wavelet algorithm had high training efficiency. Afterwards, the denoising application effectiveness of the denoising algorithm was tested. Therefore, in the standard test library MSRA1000, 1000 natural images are randomly selected and divided them into 20 groups. The denoised image is compared with the standard image to determine the similarity change. The specific algorithm application effect is shown in Fig 14.

If the similarity error value in Fig 14 is low, the quality of the image or video is good. From Fig 14, the similarity error of the Fusion algorithm proposed in this study was relatively low, in which the lowest similarity error was 0.1 and the average similarity error was 0.89. The average similarity error values of Hard TA and Soft TA were 2.63 and 1.95. This suggests that the fusion threshold algorithm performs better in preserving image or video quality. The reason may be that the fusion threshold algorithm can better maintain the integrity of image details in the denoising by integrating WT technology and threshold processing strategy. This means that the fusion algorithm can effectively reduce noise and reduce the loss of image information. In view of this, the image denoising technology combining threshold algorithm and wavelet algorithm shows excellent performance in output denoising image quality, denoising calculation speed, and algorithm stability. It can better adapt to complex and ever-changing noise environments. The algorithm has higher universality. Compared to traditional image denoising algorithms, the fusion algorithm has stronger comprehensive denoising performance and higher stability. It can better meet the current performance requirements of image denoising technology.

**Fig 15:**
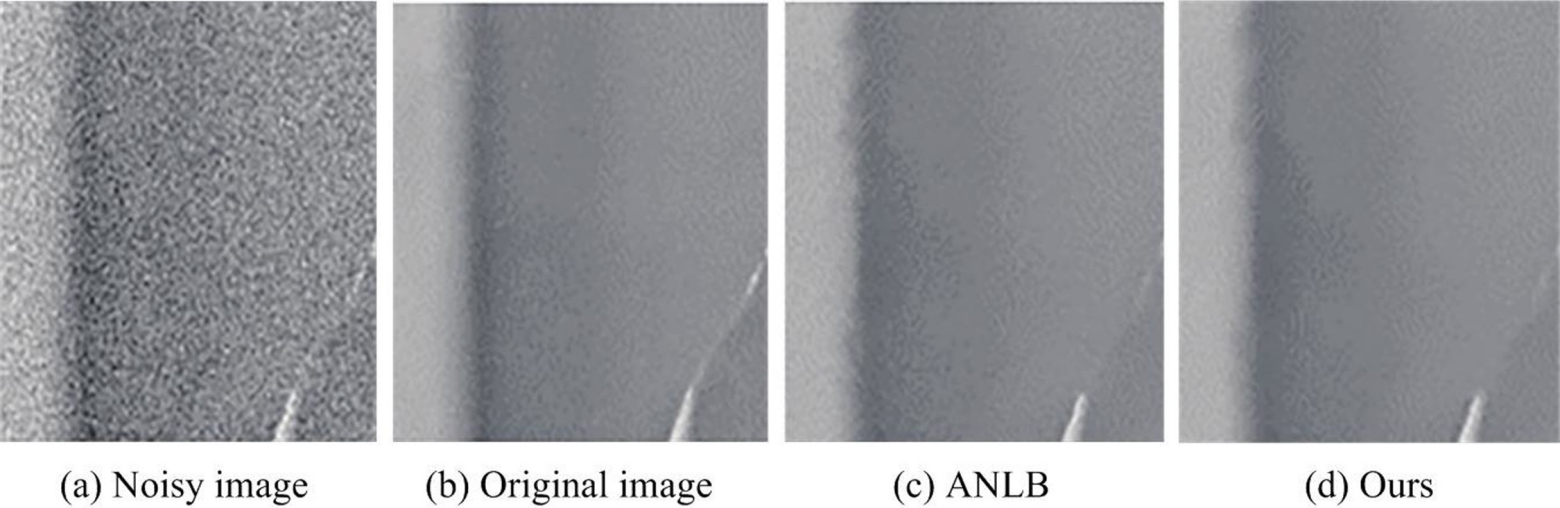
Comparison of image denoising results between two algorithms.

Finally, a random image is selected from the Lena image data set. The pink noise of σ = 20 is added to the original image. In addition, the image denoising methods (Affine Non Local Bayes, ANLB) of mixed affine and non local Bayes algorithms [25] are compared with the constructed experimental hybrid algorithm. The obtained image denoising results are shown in Fig 15.

**Fig 16:**
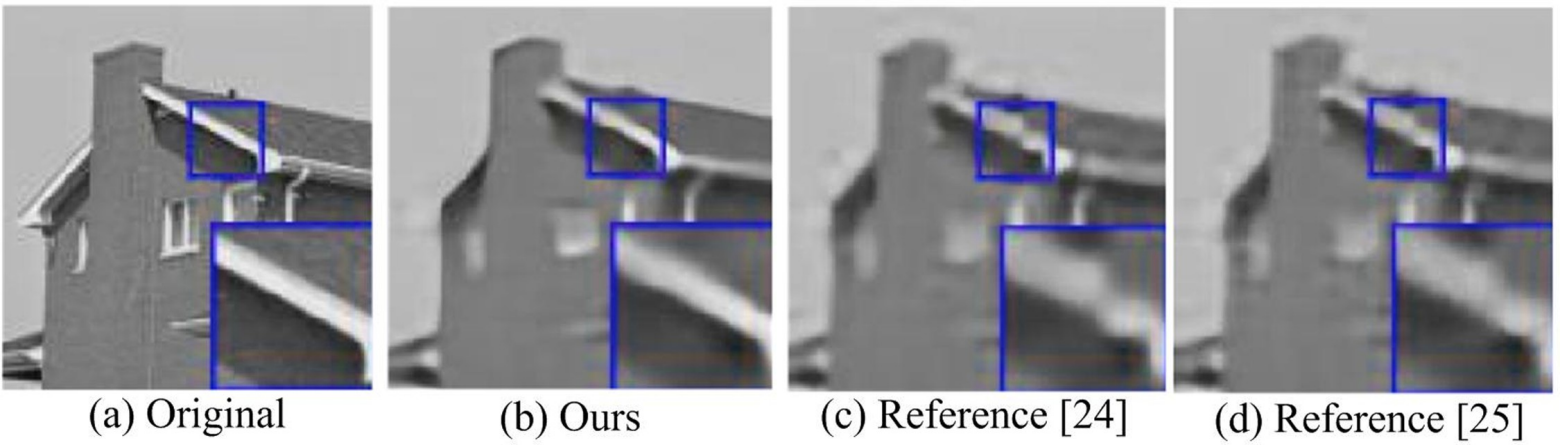
Comparison of denoising effects of different algorithms.

In Fig 15, the ANLB method produced more false textures in the flat areas of the denoising results. The edges were slightly distorted, while the hybrid algorithm had advantages in both aspects. Through subjective and objective analysis, it can be concluded that the hybrid algorithm has a better noise removal effect, which produces clearer images. Then the experimental methods in references [24, 25] were selected to compare the denoising effects with the constructed method, as shown in Fig 16 for details.

As can be seen from Fig 16, compared with the multi-level image denoising method based on wavelet transform in reference [24] and the image denoising method based on Caputo diffusion equation in reference [25], the image denoising technology based on fusion threshold algorithm and wavelet algorithm designed in this study is better in the output denoising picture quality. The reason may be that this method tries to keep the integrity of image details while removing Gaussian noise, and can achieve remarkable results in reducing mean square error and improving peak signal-to-noise ratio.

## 5. Conclusion

The purpose of image denoising is to better eliminate the noise information in the image while retaining the true signal of the image. To solve this problem, the research proposes an image denoising method based on improved threshold function and WT. The experiment combines the WT and finite ridgelet transform to jointly improve the output noise reduction performance of the image. An improved threshold function is used to reconstruct the image to improve the image quality. From the data results, after adding Gaussian noise with a variance of 0.01 to the image, the PSNR of the image obtained by the fusion algorithm increased by more than 1.10%. The MSE was significantly reduced by 0.06%. In terms of denoising running time, the fusion algorithm had the lowest running time, which was only 13ms. Compared to the Hard TA and Soft TA, it saved 9ms and 3ms respectively. In the comparison of SSIM values of different algorithms, the SSIM value of the fusion algorithm was stable at around 98.3% during the entire process. In practical applications, the image obtained through the hybrid algorithm is clearer and has less edge distortion texture. The above results show that the image texture obtained after denoising by the fusion algorithm is displayed more clearly and the denoising effect is better. However, it should be noted that the data set involved in the experiment does not cover all possible noise types. Future work will be extended to more types of noise, verifying the denoising efficiency and effectiveness of the fusion algorithm on a wider data set.

## Supporting information

S1 Dataset (DOC)
