# Supplementary material for: Image denoising method integrating ridgelet transform and improved wavelet threshold
Source: PLoS One. 2024 Sep 6;19(9):e0306706. doi: 10.1371/journal.pone.0306706 (PMC11379185; doi:10.1371/journal.pone.0306706)
Supplement: S1 Dataset — (DOC) [file pone.0306706.s001.doc]

The data in Figure 8

| PSNR/dB | RSNR-gray images | | | | |
| --- | --- | --- | --- | --- | --- |
|  | Hard threshold algorithm | Soft threshold algorithm | Mean algorithm | Median algorithm | Fusion algorithm |
| 0.01 | 74.60 | 74.75 | 75.24 | 75.59 | 76.50 |
| 0.02 | 74.41 | 74.12 | 74.58 | 74.59 | 76.01 |
| 0.03 | 74.18 | 73.77 | 74.35 | 74.30 | 75.50 |
| MSE/dB | MSE | | | | |
|  | Hard threshold algorithm | Soft threshold algorithm | Mean algorithm | Median algorithm | Fusion algorithm |
| 0.01 | 0.0021 | 0.0023 | 0.0023 | 0.0020 | 0.0014 |
| 0.02 | 0.0025 | 0.0024 | 0.0019 | 0.0020 | 0.0016 |
| 0.03 | 0.0025 | 0.0027 | 0.0024 | 0.0022 | 0.0017 |

**The data in Figure 9**

| PSNR/dB | RSNR-color images | | | | |
| --- | --- | --- | --- | --- | --- |
|  | Hard threshold algorithm | Mean algorithm | Soft threshold algorithm | Median algorithm | Fusion algorithm |
| 0.01 | 74.71 | 74.52 | 71.08 | 70.54 | 73.77 |
| 0.02 | 74.54 | 73.91 | 70.15 | 69.78 | 73.51 |
| 0.03 | 73.99 | 73.82 | 69.78 | 69.00 | 72.94 |
| MSE/dB | MSE | | | | |
|  | Hard threshold algorithm | Mean algorithm | Soft threshold algorithm | Median algorithm | Fusion algorithm |
| 0.01 | 0.0022 | 0.0031 | 0.0060 | 0.0061 | 0.0036 |
| 0.02 | 0.0024 | 0.0033 | 0.0070 | 0.0070 | 0.0039 |
| 0.03 | 0.0028 | 0.0039 | 0.0085 | 0.0081 | 0.0045 |

**The data in Figure 11**

| Recall | Local contrast | | | | | |
| --- | --- | --- | --- | --- | --- | --- |
|  | IT | GB | AC | MZ | Fusion algorithm | |
| 0.20 | 0.62 | 0.63 | 0.64 | 0.52 | 0.93 | |
| 0.40 | 0.49 | 0.60 | 0.60 | 0.48 | 0.94 | |
| 0.60 | 0.39 | 0.55 | 0.53 | 0.42 | 0.92 | |
| 0.80 | 0.30 | 0.44 | 0.42 | 0.34 | 0.87 | |
| 1.00 | 0.21 | | | | | |
| Recall | Global contrast | | | | | |
|  | LC | FT | SR | CA | RC | Fusion algorithm |
| 0.20 | 0.70 | 0.66 | 0.53 | 0.68 | 0.90 | 0.94 |
| 0.40 | 0.72 | 0.62 | 0.43 | 0.65 | 0.89 | 0.94 |
| 0.60 | 0.68 | 0.52 | 0.37 | 0.59 | 0.87 | 0.92 |
| 0.80 | 0.53 | 0.35 | 0.31 | 0.50 | 0.77 | 0.80 |
| 1.00 | 0.21 | | | | | |

**The data in Figure 12**

| Training volume/×10^2^ | SSIM (%) | | | | |
| --- | --- | --- | --- | --- | --- |
|  | Median algorithm | Fusion algorithm | Hard TA | Soft TA | Semi-Soft TA |
| 3 | 97.12 | 98.11 | 97.99 | 96.90 | 97.20 |
| 6 | 96.89 | 98.25 | 97.51 | 96.25 | 97.11 |
| 9 | 97.12 | 98.07 | 96.74 | 96.85 | 97.39 |
| Training volume/×10^2^ | MSE | | | | |
|  | Median algorithm | Fusion algorithm | Hard TA | Soft TA | Semi-Soft TA |
| 3 | 1.08 | 0.11 | 0.30 | 1.20 | 0.84 |
| 6 | 1.10 | 0.30 | 0.71 | 2.08 | 1.19 |
| 9 | 1.16 | 0.16 | 1.28 | 1.26 | 0.68 |

The data in Figure 13

| Sample number | Running time(ms) | | | |
| --- | --- | --- | --- | --- |
|  | Median algorithm | Fusion algorithm | Hard TA | Soft TA |
| 3 | 14.52 | 12.51 | 20.00 | 16.27 |
| 6 | 14.97 | 13.68 | 20.01 | 16.29 |
| 9 | 14.74 | 13.01 | 20.88 | 16.28 |

**The data in Figure 14**

| Sample number | Running time(ms) | | | |
| --- | --- | --- | --- | --- |
|  | Median algorithm | Fusion algorithm | Hard TA | Soft TA |
| 3 | 1.31 | 0.10 | 2.87 | 0.41 |
| 6 | 1.37 | 1.14 | 2.50 | 1.92 |
| 9 | 0.84 | 0.13 | 2.46 | 1.30 |
